# Non-invasive monitoring of tissue oxygenation during laparoscopic donor nephrectomy

**DOI:** 10.1186/1471-2482-8-8

**Published:** 2008-04-17

**Authors:** Nicole J Crane, Peter A Pinto, Douglas Hale, Frederick A Gage, Doug Tadaki, Allan D Kirk, Ira W Levin, Eric A Elster

**Affiliations:** 1 Naval Medical Research Center, Combat Casualty Care, Silver Spring, MD 20910, USA; 2 National Institutes of Health, National Cancer Institute, Urologic Oncology, Bethesda, MD 20892, USA; 3 University of Florida, College of Medicine, General Surgery Residency Program, Jacksonville, FL, 32209, USA; 4 Department of Surgery, National Naval Medical Center, Bethesda, MD 20892, USA; 5 Emory University Hospital, Emory Transplant Center, Atlanta, GA 30322, USA; 6 National Institutes of Health, National Institute of Diabetes and Digestive and Kidney Diseases, Laboratory of Chemical Physics, Bethesda, MD 20892, USA; 7 Department of Surgery, Uniformed Services University, University of Health Sciences, Bethesda, MD 20892, USA

## Abstract

**Background:**

Standard methods for assessment of organ viability during surgery are typically limited to visual cues and tactile feedback in open surgery. However, during laparoscopic surgery, these processes are impaired. This is of particular relevance during laparoscopic renal donation, where the condition of the kidney must be optimized despite considerable manipulation. However, there is no *in vivo *methodology to monitor renal parenchymal oxygenation during laparoscopic surgery.

**Methods:**

We have developed a method for the real time, *in vivo*, whole organ assessment of tissue oxygenation during laparoscopic nephrectomy to convey meaningful biological data to the surgeon during laparoscopic surgery. We apply the 3-CCD (charge coupled device) camera to monitor qualitatively renal parenchymal oxygenation with potential real-time video capability.

**Results:**

We have validated this methodology in a porcine model across a range of hypoxic conditions, and have then applied the method during clinical laparoscopic donor nephrectomies during clinically relevant pneumoperitoneum. 3-CCD image enhancement produces mean region of interest (ROI) intensity values that can be directly correlated with blood oxygen saturation measurements (R^2 ^> 0.96). The calculated mean ROI intensity values obtained at the beginning of the laparoscopic nephrectomy do not differ significantly from mean ROI intensity values calculated immediately before kidney removal (*p *> 0.05).

**Conclusion:**

Here, using the 3-CCD camera, we qualitatively monitor tissue oxygenation. This means of assessing intraoperative tissue oxygenation may be a useful method to avoid unintended ischemic injury during laparoscopic surgery. Preliminary results indicate that no significant changes in renal oxygenation occur as a result of pneumoperitoneum.

## Background

In the past 10 years the use of living donor kidneys have markedly increased and in 2003 surpassed deceased donors as the predominant source of donor organs [1]. Laparoscopic donor nephrectomy has become a major driving force in increasing the acceptance of living donation. Laparoscopic donor nephrectomy (LDN) is thought to have several potential advantages over open donor nephrectomy (ODN) [1,2]. Namely, laparoscopic procedures require a shorter hospital stay, decreased amounts of analgesia, allow for a faster return to work and provide improved cosmesis. However, disadvantages of laparoscopic surgery include slightly longer warm ischemic times, and increased incidences of delayed graft function, the later thought to be the result of tissue hypoxia from pneumoperitoneum associated hypoperfusion and organ manipulation [1,2]. These issues, while minor in most donors, are increasingly problematic in situations utilizing older donors, or organs intended for use in very small children [3,4]. Many technical aspects of laparoscopic donation have been developed to minimize organ ischemic injury, and several parameters have been monitored to indirectly assess the general tolerance of pneumoperitoneum, including cardiac output, stroke volume, mean arterial pressure, urine output, systemic vascular resistance and end-tidal CO_2_. All of the methodologies are limited by their inability to assess the organ directly. The most direct measurement would be that of whole organ oxygenation. Unfortunately, to date there has not been a method to evaluate tissue oxygenation laparoscopically in a time frame that is clinically relevant. The ability to intraoperatively monitor renal paranchyemal oxygenation would be useful in a number of clinical situations in which prompt resolution may have a dramatic effect. One such an example is encountered when during the course of the operation the blood supply to the organ becomes impaired by the technical manuevers done during dissection (i.e., approaching the vessels from the posterior aspect). Prompt recognition of decreased oxygenation would allow for repositioning of the kidney and re-establishment of blood flow. Other examples include the determination of secondary renal arteries and the establishment of a baseline acceptable pneumoperiotenum, potentially useful in older donors.

In this report, we describe the development of a means of directly assessing organ oxygenation during laparoscopic surgery. Spectroscopic information obtained by a standard 3-CCD camera used in laparoscopic surgery is processed thereby providing real time feedback to the surgeon using equipment readily available in any standard laparoscopic operating suite.

## Methods

### Algorithm: 3-CCD Detector Analysis

**Figure 1:**
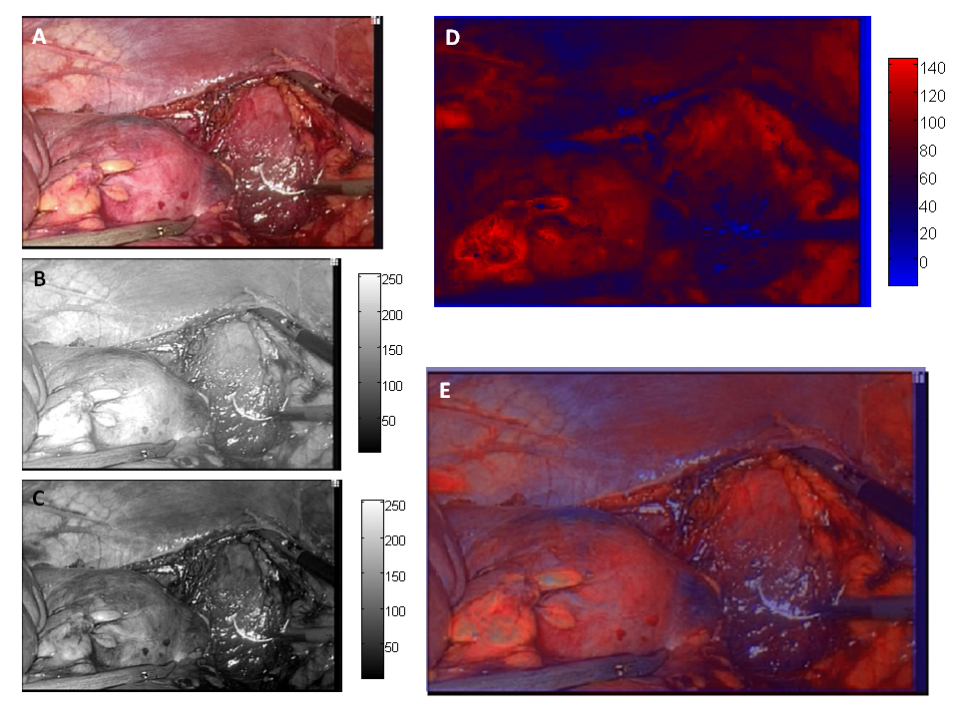
**Laparoscopic donor nephrectomy.** (a) Image of kidney extracted from DVD as a .tiff file. (b) Red CCD response, plotted in grayscale. (c) Blue CCD response, plotted in grayscale. (d) Red CCD response minus blue CCD response, set to special color scale. (e) Calculated CCD response image overlaid onto the original image at 55% transparency.

Images of porcine nephrectomies and human LDNs were used to calculate mean intensity values from the 3-CCD camera as described previously [5,6]. Briefly, the human nephrectomies were recorded using a Storz laparoscopic tower (Tuttlingen, Germany), equipped with a 3-CCD camera. The porcine nephrectomies were recorded using an Olympus laparoscopic tower (Orangeburg, NY, USA) coupled to a Stryker 3-CCD camera (San Jose, CA, USA) without the laparoscope attachment. Individual frames of the recorded video were extracted as TIFF (tagged image format file) files as seen in Figure 1a. Using Matlab software (Natick, MA, USA) the blue CCD response (Figure 1c) was subtracted from the red CCD response (Figure 1b) [6]. This difference has been directly correlated with the spectral response of hemoglobin in both the blue and red regions of the visible spectrum [6]. The resulting image (Figure 1d) is plotted in a modified color scale. An intense red color indicates pixels receiving the most signal from the red CCD and an intense blue color indicates pixels receiving the least amount of signal from the red CCD. Finally, the calculated image is overlaid onto the original TIFF image (Figure 1e), allowing complete visual registry along with enhancement.

### Testing: Validation using a Porcine Model

Porcine experiments (n = 4) were performed as a validation of the sensitivity and correlation of 3-CCD mean intensity values with actual blood and tissue oxygenation. Porcine laparotomies, as part of an animal protocol approved by the Institutional Animal Care and Use Committee, were used to assess the extent of ischemic injury (i.e. decreased tissue oxygenation) incurred by reduced fractions of inspired oxygenation (FiO_2_) during surgery. Standard open surgical techniques were employed to exposure the kidney and renal hilum. Mean region of interest (ROI) values were calculated from the video images of the surgery collected using the 3-CCD camera and compared to measured arterial and venous oxygen saturation values (saO_2 _and svO_2_).

**Figure 2:**
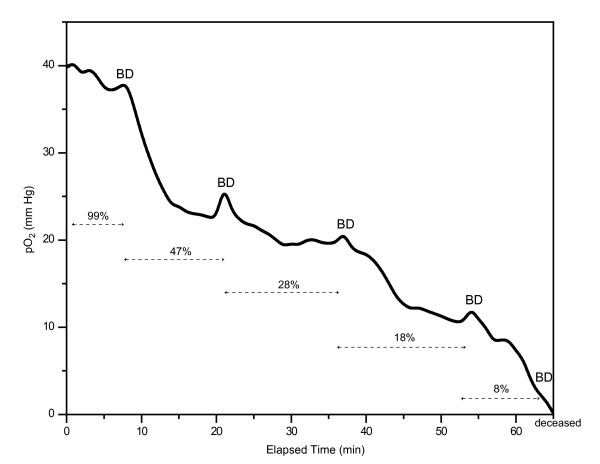
**Renal parenchymal oxygen tension (pO_2_) measured via a fluorescence probe, as the fraction of O_2 _(FiO_2_) is reduced over 65 minutes.** The FiO_2 _is indicated with dashed arrows. The times of blood draws, where blood gas measurements were made (sO_2_), are indicated by BD.

The 3-CCD camera and the tower light were mounted to the overhead operating room light such that both kidneys were evenly illuminated and in the field of view. The fraction of inspired oxygenation was decreased incrementally (~100%, ~50%, ~30%, ~20%, 9%) during the determination. After each decrease in FiO_2_, the kidney was allowed to equilibrate for approximately 15 minutes. Renal oxygen tension (pO_2_) was measured directly by an OxyLite fluorescence needle probe (Oxford Optronics Ltd., Oxford, UK) in the superior pole of the kidneys (Figure 2). Fluorescence measurements confirm changes in blood oxygen saturation in the kidney itself as a result of reduced FiO_2_. Blood was also drawn from the aorta and renal veins following equilibrium and immediately analyzed for saO_2 _and svO_2 _using a portable blood gas analyzer (iStat, Abbott Point of Care Inc., East Windsor, NJ, USA).

For each different FiO_2 _level, mean values for the ROIs in the images were calculated from rectangles comprised of 900–4,500 pixels. While rectangle sizes varied for each image, the dimensions of a rectangle at a particular location in the image remained relatively consistent from image to image; the orientation of the kidney changed little throughout the determination.

### Implementation: Laparoscopic Donor Nephrectomy

Healthy renal donors (n = 9) were enrolled in a National Institutes of Health Institutional Review Board (NIH IRB) approved protocol to assess outcomes during and after living donor nephrectomy as well as one of several NIH IRB approved protocols to assess allograft function. LDN was performed using previously described techniques with a continuous pneumoperitoneum of 15 mmHg [7]. Renal allografts were then transplanted using standard surgical techniques. All nine kidneys were left kidneys, with a single artery, vein, and ureter, which were immediately flushed with cold University of Wisconsin solution prior to transplantation. Donor and recipient demographics are outlined in Table 1.

**Table 1:** Patient Demographics

**Case**	**Donor Age (yrs)**	**BMI (kg/m**^2^**)**	**Gender**	**OR time (min)**	**EBL (ml)**	Fluid (ml)*	**Urine (ml)**
1	48	26.01	female	300	700	8600	1400
2	49		female	240	400	6200	3000
3	27	23.20	female	220	100	6000	1400
4	53	26.23	male	300	300	6600	1900
5	39	26.79	male	240	400	8500	2400
6	26	23.30	male	340	< 50	4400	1100
7	42	37.08	female	280	150	3700	900
8	28	22.74	female	355	200	5800	1400
9	22	20.69	male	385	100	5800	1665
*Mean*	*37.1 ± 11.6*	*25.76 ± 5.03*		*296 ± 156*	*294 ± 204*	*6178 ± 1662*	*1685 ± 660*

BMI – body mass index; OR – operating room; EBL – estimated blood loss

* Fluid was lactated ringers in all cases.

For each case and time series of extracted frames, mean values for the ROIs in the images were calculated from rectangles containing at least 625–44,000 pixels. Rectangle size was not consistent image to image because the orientation of the kidney was constantly changing. Glare proved troublesome by contributing false blue regions in the subtracted images; thus, regions of glare were neglected when calculating mean values for the ROIs prior to normalization.

### Statistical Method

The student's t-test was used to determine significant differences between ROI mean values. Means were considered significantly different with *p*-values less than 0.05. For comparisons of mean ROI values determined during the pig nephrectomies, a paired t-test for sample means was applied. For comparisons of mean ROI values calculated within the same human surgical case, an unpaired, two-tailed t-test with equal variances was applied.

## Results

### Spectroscopic Evidence

#### 1. Animal Model

The described technique was first tested and validated using a porcine model. A fluorescent needle probe (OxyLite) was used to monitor changes in renal oxygenation, alongside 3-CCD assessment. In Figure 2, the solid line follows pO_2 _levels as the FiO_2 _level, indicated over the duration of the dashed arrows, is decreased. Figure 2 demonstrates a drop in the pO_2 _in the kidney each time the percentage of inspired oxygen is decreased, followed by a region of little change (equilibration). Each venous blood draw (BD) is marked by a small increase in pO_2_; as the blood is drawn from the renal vein, fresh blood flows from the renal artery into the kidney, creating a temporary increase in tissue oxygenation.

**Figure 3:**
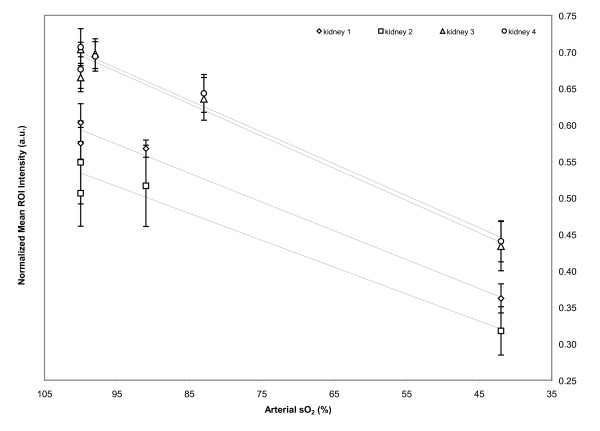
**Linear relationship of calculated mean normalized ROI (region of interest) values and measured venous sO_2 _as the FiO_2 _is decreased from 100% to 8% in four different kidneys.** R^2 ^= 0.9722 (○), 0.9854 (□), 0.9624 (△) and 0.9801 (◇).

At 100% inspired oxygen, saO_2 _is 100% with mean ROI values from the detector data of 0.66 ± 0.02, 0.68 ± 0.03, 0.60 ± 0.03, and 0.51 ± 0.05, for kidneys 1 through 4. Similar mean ROI values were observed for ~50% FiO_2 _with 100% saO_2 _(0.70 ± 0.01, 0.71 ± 0.03, 0.57 ± 0.02, and 0.55 ± 0.06, for kidneys 1 through 4). For kidneys 1 and 2, at 28% FiO_2 _and an saO_2 _of 98%, the mean ROI values calculated were 0.70 ± 0.02 and 0.69 ± 0.02, still largely unchanged from 100 and 50% FiO_2_. Similar values were observed for kidneys 3 and 4 at 21% FiO_2 _and an saO_2 _of 91% (0.57 ± 0.01 and 0.52 ± 0.06). Though the drop in FiO_2 _from ~50% to 28% and 21% is significant, FiO_2_'s of 28% and 21% are similar to room air; thus, a large drop in mean ROI values from ~50% FiO_2 _to 28% and 21% FiO_2 _is not expected. There is, however, a definite decrease in the mean ROI values determined for kidneys 1 and 2 with an FiO_2 _of 18% and saO_2 _of 83% (0.64 ± 0.03 for both kidneys). The mean ROI values drop significantly when the FiO_2 _is decreased to ~9% (0.43 ± 0.03, 0.44 ± 0.03, 0.36 ± 0.02, and 0.32 ± 0.03, for kidneys 1–4) when compared to previous mean ROI values (*p *= 0.005). When the calculated ROI values are plotted against the saO_2 _for each kidney, there is a clear linear relationship between ROI values and saO_2 _measurements, as indicated by Figure 3.

#### 2. Role of Pneumoperitoneum

**Figure 4:**
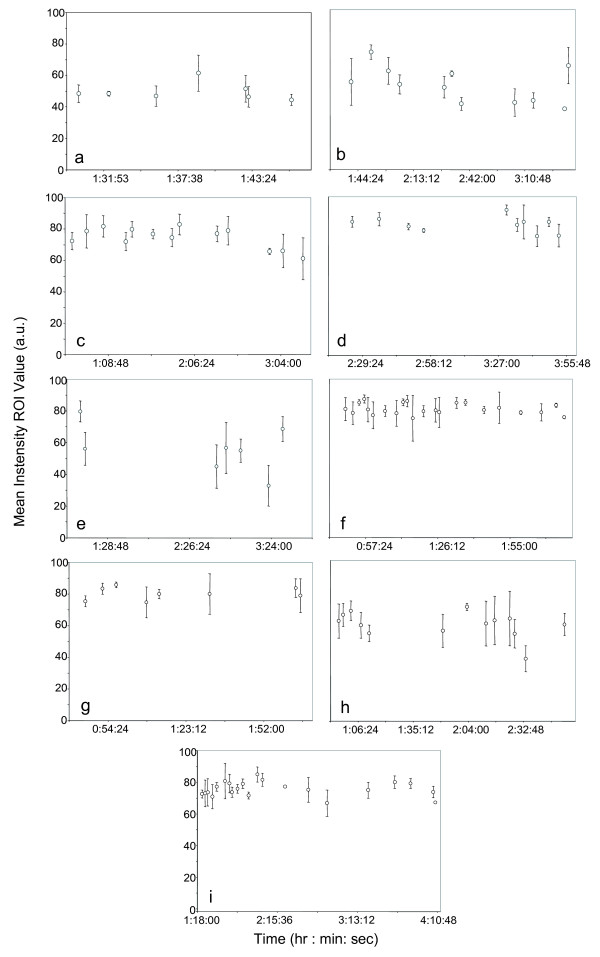
The normalized mean ROI values for nine donor laparoscopic nephrectomies: (a-i) case 1–9, respectively.

This technique was then applied to nine human LDN cases to determine if pneumoperitoneum reduced parenchymal oxygenation. Interval monitoring of kidneys showed stable oxygenation without evidence of significant hypoxia. The mean values for the ROIs are presented chronologically for each case in Figure 4. Various time points are examined, where most gaps in the sampling intervals were less than 15 minutes. For case 5, however, unambiguous image data of the kidney was not obtainable for a period of approximately 95 minutes. The duration over which image frames were collected also varied. Case 1 sampled the shortest period, with a duration of ~16 minutes, while case 9 has the longest sampling duration, ~170 minutes. Cases 1 through 9 appear to fluctuate slightly but not significantly, in spite of differing normalized ROI mean intensities. The normalized ROI mean intensity values for case 5 decreased over time with respect to the starting point (79.78 ± 6.62 versus 56.20 ± 10.44, 44.98 ± 13.71, 56.67 ± 16.05, 55.07 ± 7.24, 32.84 ± 12.76), but returned to a comparable value by the end of the sampling period (79.78 ± 6.62 versus 68.64 ± 7.83).

Table 2 displays the mean starting ROI values which are compared to the mean ending ROI values for each case with the corresponding *p*-values. No statistically significant differences exist between the starting and the ending mean ROI values for all cases, with *p*-values all greater than 0.05. The mean starting ROI value for all cases, 70.21 ± 12.36, is comparable to the mean ending ROI value for all cases, 66.43 ± 10.53 (*p *= 0.49). The small fluctuation in values implies that the oxygenation of the kidney is relatively stable with an intraabdominal pressure of no more than 15 mm Hg. Note, intrapatient comparison of mean ROI values is not performed due to variability in abdominal illumination from case to case and variability in duration of pnuemoperitoneum from case to case.

**Table 2:** Mean intensity normalized ROI values. Mean intensity normalized ROI values of both the start and end time points for each case. All *p*-values are above 0.05 and indicate the mean ROI values for the start and end time points are not significantly different.

Case	Mean ROI	σ*	Mean ROI	σ*	*p-value*
	*Starting point*		*End point*		

1	48.40	5.60	44.48	3.55	*0.13*
2	54.88	14.56	65.02	11.31	*0.56*
3	72.42	5.51	61.17	13.38	*0.16*
4	84.27	3.38	75.58	7.15	*0.14*
5	79.78	6.62	68.64	7.83	*0.21*
6	81.17	7.24	75.98	0.97	*0.38*
7	75.50	3.37	78.96	10.62	*0.60*
8	62.41	10.58	60.29	6.90	*0.79*
9	73.09	2.49	67.74	0.19	*0.07*

* σ = one standard deviation

While not presented in this manuscript, we have also examined laparoscopic partial nephrectomies, where complete hilar clamping or renal arterial clamping is performed, and so, we have explored variation in oxygenation as a direct result of surgically-induced vasoconstriction. We do in fact see the mean ROI values decrease after clamping and then return to baseline ROI values after reperfusion (*p *≤ 0.05 in all cases).

### Clinical Findings

All spectroscopic evidence for lack of change in kidney oxygenation during pneumoperitoneum is supported by standard clinical methods for assessing kidney function. Immediate graft function was seen in all recipients. The mean one day pre-operative donor serum creatinine level is 0.9 ± 0.2 mg/dl. The mean post-operative recipient serum creatinine levels for post-operative days 1, 5 and 20 were 5.2 ± 1.6 mg/dl, 1.6 ± 0.4 mg/dl, and 1.5 ± 0.4 mg/dl, indicative of brisk post transplant function. Individual case values are shown in Table 3. The mean post-operative recipient BUN levels, considered normal between 8 and 20 mg/dl, (shown in Table 4) for post-operative days 1, 5 and 20 are 36 ± 13 mg/dl, 25 ± 8 mg/dl, and 17 ± 5 mg/dl. With the exception of case 9, the recipient serum creatinine levels and recipient BUN levels were all within normal limits by post-operative day 20.

**Table 3:** Donor and recipient serum creatinine levels (pre- and post-operative). Normal serum creatinine levels are ≤ 1.6 mg/dl.

**Case**	**Donor Serum Creatinine (mg/dl)**	**Recipient Serum Creatinine (mg/dl)**		
	Pre-op day 1	Post-op day 1	Pre-op day 5	Post-op day 10

1	0.8	5.1	1.5	1.7
2	0.7	5.1	1.7	1.6
3	0.7	7.9	1.8	1.6
4	0.7	5.6	1.2	1.0
5	1.2	4.1	1.1	0.9
6	1.0	3.6	1.3	1.3
7	0.8	4.1	1.4	1.6
8	0.7	7.9	2.4	1.7
9	1.1	3.8	1.9	2.0

**Table 4:** Recipient blood urea nitrogen values at post-operative days 1, 5 and 20. Normal BUN is 8–20 mg/dl.

**Case**	**Recipient Blood Urea Nitrogen (mg/dl)**		
	*Post-op day 1*	*Post-op day 5*	*Post-op day 20*

1	30	27	15
2	34	23	16
3	53	35	20
4	27	17	15
5	58	21	12
6	22	14	19
7	35	20	15
8	22	38	15
9	45	27	28

## Discussion

This manuscript reports, for the first time, the use of a 3-CCD camera to detect hypoxia *in vivo *and in doing so, introduces a potential means of avoiding unintended hypoxic injury during laparoscopic surgery. In addition to demonstrating that tissue oxygenation is not impaired during routine donor nephrectomy, the study highlights a novel and useful method for intraoperative functional imaging. As such, this study supports current laparoscopic methodology with CO_2 _pneumoperitoneum and introduces a method with broad potential as an intraoperative monitoring device.

We chose the open porcine model so that pneumoperitoneum would not be a variable when considering the effect of blood oxygenation on the mean ROI values calculated from the 3-CCD camera. While the equipment employed in this study is used in an open fashion, it is designed for laparoscopic incorporation. An obvious model for altered tissue oxygenation would have been clamping the renal hilum; however, in our hands, it has been extremely difficult to partially clamp the hilum in a controlled fashion (progressive hilar clamping) or to allow the kidney to reperfuse in a controlled and partial manner. Without being able to control the tissue oxygenation or deoxygenation, we could not reliably collect enough data points for a clear correlation. Thus, the decreasing FiO_2 _model was chosen for our validation experiment. This model directly enabled correlation of oxygen delivery with 3-CCD mean ROI values.

Outside of clinical studies or animal models, intraoperative assessment of tissue oxygenation during LDN is currently limited to visualization of the parenchyma with the naked eye. Although kidney function, in general, has been monitored by creatinine and/or tissue pathology, results from both techniques are not obtained immediately. Further, the use of these assays requires a significant amount of time following injury. In general, as a result of technical variables, laparoscopic nephrectomies require a longer warm ischemia time than open nephrectomies [8], which may result in additional ischemia as a result of the pneumoperitoneum [9]; thus, an evaluation of the kidney's viability becomes vital. It is clearly beneficial to be able to monitor the status of the kidney in real time.

Current techniques to assess the kidney during surgery include a non-contact laser tissue blood flowmeter (NCLBF) [10], pulse oximetry, fluorescein, laser autofluorescence imaging [11], measuring erythrocyte velocity [12], and fluorescence oxygen tension measurements [13,14]. In a study by Ando and coworkers, NCLBF was compared with pulse oximetry and fluorescein for the assessment of ischemic tissue. It was determined that NCBLF outperformed pulse oximetry and fluorescein in accuracy and sensitivity in predicting the viability of ischemic bowel [10]. Pulse oximetry and fluorescein have a high risk of failure for detecting tissue necrosis in addition to a poor accuracy rate for evaluation purposes. The disadvantage of a technique like NCLBF is that the measurement is made via a pencil probe, appropriate for open surgery but not laparoscopic surgery. Laser autofluorescence imaging operates on the assumption that autofluorescence changes with 335 nm excitation are attributed to NADH which accumulates in tissue during ischemia [11]. While the technique shows promising results and allows for real time in vivo imaging, this method requires special instrumentation and is not easily converted to a format for use with a laparoscopic tower. Measurement of erythrocyte velocity using a magnifying endoscope [12] is the only above-mentioned technique that could be easily applied during a laparoscopic surgery. However, the pencil-lens probe of the endoscope samples and evaluates only a small portion of the tissue during a single measurement. Evaluation of the kidney as a whole would require a large number of sampling points, proving inefficient in a time limited scenario. While the OxyLite probe is very effective for sampling tissue oxygenation within the tissue itself [13,14], it suffers from the same limitations as the erythrocyte velocity measurements; the needle probe allows for only spot measurements, not global tissue oxygenation measurements. Thus, currently, there are no techniques that are well suited for the diagnosis of tissue viability during laparoscopic surgery.

The technique presented in this manuscript presents real-time capability with straight forward incorporation of additional software and computer interfacing. Real-time function will not require additional hardware or equipment that is not readily available in the operating suite. While glare proves troublesome for some images, inclusion of polarizing optics directly into the laparoscope itself would obviate this problem. Though the technique probes the surface of tissue and is not able to detect tissue oxygenation beneath fatty regions, as in LDN, this obstacle is typically negated by the need for surgical dissection. Similarly, bloody operating fields are only problematic if the entire region of interest is obscured; several small, exposed regions are sufficient for calculating mean ROI values. In addition, surface assessment of tissue oxygenation appears to reflect whole organ oxygenation as evidenced by the large animal data presented herein.

## Conclusion

We present preliminary results for a technique that has the potential to diagnose tissue ischemia in real time and in an organ specific manner during laparoscopic surgery. In addition, using this technique we demonstrate that sustained pneumoperitoneum at standard levels (15 mmHg) in a small case series did not have adverse effects on tissue oxygenation as measured by this methodology and substantiated by the clinical course. Although the calculations presented in this study were performed outside of the laparoscopic system, efficient programming will allow automatic, real time incorporation of the calculation to the laparoscopic images. While the clinical utility of this method is still unknown, we recognize that an expanded study, involving a significantly greater number of cases than presented here, would allow the development of a training set by which threshold values for tissue end-point resuscitation could be established. The developed training set would be the foundation for a clinical validation study. Another consideration for the clinical validation would be the addition of a range of pneumoperitoneum pressures, including pressures greater than 15 mm Hg. Furthermore, this technique may be broadly applicable to provide an indicator of organ ischemia during all laparoscopic surgeries.

## Competing interests

The author(s) declare that they have no competing interests.

## Authors' contributions

NJC and EAE conceived the study. IWL participated in the study design. NJC developed the technique and performed all data analysis. NJC and EAE drafted the manuscript. PAP, DH, ADK, and EAE performed the surgeries. FG and DT participated in study coordination. All authors read and approved the final manuscript.

## Pre-publication history

The pre-publication history for this paper can be accessed here:


